# Human Mesenchymal Stem Cell Therapy and Other Novel Treatment Approaches for Premature Ovarian Insufficiency

**DOI:** 10.1007/s43032-021-00528-z

**Published:** 2021-05-06

**Authors:** Mara Ulin, Esra Cetin, Elie Hobeika, Rishi Man Chugh, Hang-Soo Park, Sahar Esfandyari, Ayman Al-Hendy

**Affiliations:** 1 The University of Illinois at Chicago, Chicago, IL USA; 2 The University of Chicago, Chicago, IL USA; 3 Fertility Centers of Illinois, Glenview, IL USA

**Keywords:** Mesenchymal stem cells (MSC), Regenerative medicine, Premature ovarian insufficiency, Infertility

## Abstract

Premature ovarian insufficiency (POI) is a condition characterized by amenorrhea, hypergonadotropic hypogonadism, estrogen deficiency, and reduced follicle counts leading to infertility under the age of 40. POI occurs in approximately 1-3% of women in the general population. Evaluation is warranted when the diagnosis of POI is made to rule out underlying etiologies, which could be multifactorial. This review serves to cover the novel treatment approaches reported in the literature.

## Introduction

Premature ovarian insufficiency (POI) is a condition characterized by loss of ovarian function before the age of 40. It clinically manifests as a hypergonadotropic hypogonadic state inducing primary or secondary amenorrhea, infertility, and signs of estrogen deficiency. The incidence of POI is age specific, affecting 1 in 250 women by the age of 35 and 1 in 100 by the age of 40 [1]. Diagnosis is confirmed based on elevated follicle-stimulating hormone levels (>25 IU/L) on two occasions 1 month apart, along with low estradiol levels (<50 pg/mL) and amenorrhea for at least 4 months in women younger than 40 [2, 3]. Evaluation is warranted when the diagnosis of POI is made to rule out underlying etiologies. Many times, POI is iatrogenic, caused by surgery, chemotherapy, or radiation therapy. Other etiologies of spontaneous POI include Turner syndrome or variants of Turner mosaic, fragile X syndrome, and autoimmune disorders. In many cases, the etiology remains unknown [4, 5].

## Pathophysiology

Follicles are the structural units of the ovary, comprising a single oocyte surrounded by supporting somatic cells. Folliculogenesis starts in fetal life with primordial follicle development, the majority of which will remain dormant. Folliculogenesis is a complex phenomenon that starts with the initial recruitment and activation of a primordial follicle. After activation, primordial follicles grow and mature into primary, secondary, and finally antral follicles. The mechanism underlying the developmental progression of human primordial follicles is unclear. However, the initial recruitment has been linked to protein kinase B (Akt) and mammalian target of rapamycin (mTOR) signaling pathways [6]. Follicles can be found in a preantral (primordial, primary, and secondary) or antral stage (tertiary, preovulatory, and ovulatory, also called Graafian). In the preantral phase, autocrine and paracrine mechanisms play a role in follicle development, primarily through growth factor signaling; little is known about hormone signaling in the gonadotropin-independent stage. Lasting several months, this phase involves follicle growth from the primordial stage to the antral stage. After this point, most antral follicles undergo atresia, with very few reaching the preovulatory stage. In the antral stage, follicles gain gonadotropin sensitivity and respond to follicle-stimulating hormone (FSH), the levels of which increase at the beginning of every menstrual cycle [7]. Estrogen production by the growing follicle increases and is the main regulatory negative feedback between the ovary and anterior pituitary. When estrogen levels exceed 200 pg/mL, regulatory feedback becomes positive, inducing a surge of luteinizing hormone (LH) that initiates ovulation and meiotic divisions of the oocyte [8].

The number of follicles in the ovarian cortex decreases from ~6 million in a female fetus at 22 weeks of gestational age to ~1 to 2 million at birth. This supply lasts until she reaches her fourth and fifth decade of life. During each ovulatory cycle, the number of follicles decreases eventually, leading to the onset of menopause or cessation of her ovulatory function [9]. In each ovulation cycle, the ovary losses around 1000 follicles in the process of selecting a dominant follicle [8]. It is estimated that only 400 follicles will ovulate and have a reproductive potential during a woman’s lifetime, while the remaining follicles undergo atresia. A wide variation exists in the number of eggs between women of any age [10]. By the time spontaneous menopause is reached, approximately 1000 follicles remain in the ovary. Debate is ongoing as to whether women are born with a fixed number of oocytes established at birth or if their ovarian germline stem cells can differentiate into oocytes [9, 11, 12].

Whether in the setting of POI, or even spontaneous menopause, there remains a small pool of primordial follicles present in the ovarian cortex. Due to the delay in childbearing in both underdeveloped and developed countries, many women develop POI or enter menopause without having completed childbearing. For these women, if they did not bank oocytes or embryos earlier in life, the only options for building a family are the use of donated oocytes or adoption, which are associated with significant cost, psychological impact, and ethical challenges [12]. Moreover, they may be discouraged and even forbidden in certain religious groups or societies [13]. For women with POI, extensive research is focusing on how to recruit their remaining follicles, activate them, and enable their reproductive potential. This review describes various novel approaches currently being pursued to achieve this aim.

## Stem Cell Therapy

Stem cells are distinctive pluripotent cells that have the ability to differentiate into any cell type under the appropriate conditions. Researchers have investigated their potential use as precursors for new follicular units, or whether they can be used to activate the remaining primordial follicles in the ovarian cortex to regain reproductive potential (Tables 1 and 2).

**Table 1:** A summary of the treatment options, molecular mechanism, and biological effect in the ovary

Treatment option	Molecular mechanism	Biological effect in ovary
Reactivation of dormant follicles	PTEN inhibition PI3K activation (Li et al. 2010 [49])	Induction of follicular growth (Li et al. 2010 [49])
Amniotic stem cells	Inflammatory cytokines↓ FSH ↓ AMH↑ Estrogen↑ Hyaluronic acid synthase↑ (Liu et al. 2019 [14]; Zhang et al. 2017 [15])	Prevention of follicular atresia Amelioration of tissue injury through anti-inflammatory effects (Liu et al. 2019 [14]; Xiao et al. 2014 [16])
Umbilical cord stem cells	Angiogenesis↑ FSH↓ AMH↑ FOXO3a↑ Mohamed et al. 2019 [17]; Yang et al. 2019 [18]; Ding et al. 2018 [19])	Proliferation of granulosa cells Induction of follicle growth and development (Mohamed et al. 2019 [17]; Yang et al. 2019 [18]; Ding et al. 2018 [19])
Human placenta stem cells	T cell regulation (PI3K/Akt pathway) TGFβ↑ IFN-γ ↓ Estrogen↑ FSH↓ (Yin et al. 2018 [35])	Increased blood flow in the ovary Inhibition of apoptosis Suppression of inflammation (Yin et al. 2018 [35])
Human-derived menstrual blood cells	FGF2↑ Estrogen↑ FSH↓ (Wang et al. 2017 [20])	Induction of cell proliferation Stimulation of regeneration Inhibition of apoptosis and fibrosis (Wang et al. 2017 [20])
Adipose stem cells	Cytokines↑ Gene expression (Angpt1↑, Zcchc11↑) (Sun et al. 2013 [40])	Inhibition of granulosa cell apoptosis Increased number of follicles and oocytes Induction of angiogenesis (Sun et al. 2013 [40])
Bone marrow stem cells	Estrogen↑ AMH↑ Inhibin A↑ FSHR↑ Angiogenesis↑ (PI3K/Akt pathway) (Mohamed et al. 2018 [21]; Park Soo et al. 2019 [45])	Prevention of follicular atresia Induction of angiogenesis (Park Soo et al. 2019 [49])

**Table 2:** Clinical outcomes of current POI treatments

Treatment	Method	Number of patients recruited	Outcome
Traditional in vitro activation (Kawamura et al. 2013 [22])	Laparoscopic unilateral ovary fragmentation followed by Akt in vitro stimulation for 2 days with subsequent transplantation of the ovary fragments in the contralateral ovary (two surgeries)	27 POI	2 pregnancies
Simplified in vitro activation (Kawamura et al. 2020 [52])	Laparoscopic partial or complete cortex removal and unilateral fragmentation, followed by transplantation of the ovary fragments in the contralateral ovary (one surgery)	11 POR/DOR	4 pregnancies
Transplantation of UCMSC on collagen scaffold (Ding et al. 2018 [19])	Intraovarian injection	14 POF	2 pregnancies
Autologous BMSC therapy (Igboeli et al. 2020 [46])	Laparoscopic intraovarian injection	2 POF	No pregnancy reported
Autologous BMSC therapy (Edessy et al. 2016 [47])	Laparoscopic intraovarian injection	10 POF	1 pregnancy
Autologous BMSC therapy (Gupta et al. 2018 [23])	Laparoscopic intraovarian injection	1 POF	1 pregnancy
Autologous BMSC therapy (Gabr et al. 2016[48])	Laparoscopic intraovarian artery injection	30 POF	1 pregnancy
Autologous BMSC therapy (Herraiz et al. 2018 [24])	Intraarterial catheterization to intraovarian artery (after 5 days of stem cell mobilization to peripheral blood and apheresis)	15 POR	5 pregnancies
Autologous PRP treatment (Sills et al. 2018 [25])	Non-surgical transvaginal ultrasound-guided intraovarian injection	4 POR	1 pregnancy
Autologous PRP treatment (Pantos et al. 2019 [26])	Non-surgical transvaginal ultrasound-guided bilateral intraovarian injection	3 POF	3 pregnancies
Autologous PRP treatment (Sfakianoudis et al. 2019 [63])	Non-surgical transvaginal ultrasound-guided bilateral intraovarian injection	3 POR	3 pregnancies
Autologous PRP and gonadotropin combination treatment (Hsu et al. 2020 [27])	Non-surgical transvaginal ultrasound-guided bilateral intraovarian injection	1 POI	1 pregnancy
Autologous PRP treatment (Cakiroglu et al. 2020 [66])	Non-surgical transvaginal ultrasound-guided intraovarian injection	510 POR	43 pregnancies
Autologous high-dose PRP treatment (Petryk et al. 2020 [67])	Laparoscopic-assisted technique intraovarian injection	38 POR	10 pregnancies

### Amniotic Membrane Stem Cells

The amnion is a membrane that covers the developing embryo and keeps it contained within the amniotic fluid. It is considered a waste product that is commonly discarded after delivery; however, human amniotic stem cells (hAMSC) can be isolated from this membrane. hAMSC have been shown to be auto-regenerative, have multiple differentiation abilities, and provide anti-inflammatory effects similar to other multipotent mesenchymal stem cells (MSC) [28]. hAMSC secrete human growth factor, insulin-like growth factor-1 (IGF-1), vascular endothelial growth factor (VEGF), growth differentiation factor-9 (GDF-9), leukemia inhibitory factor (LIF), and other stem cell factors, the majority which have tissue healing properties [29]. Liu et al. demonstrated that transplantation of hAMSC in POI-induced mice decreases inflammatory cytokines, suggesting a resolution of tissue injury. These mice also showed an increase in estrogen and a decrease in FSH, confirming the resumption of ovarian function that resulted in successful pregnancies. Along with these remarkable findings, researchers noted an increase in protein levels of FSH receptor (FSHR), VEGF, IGF-1, tumor necrosis factor alpha (TNF-α), and interleukin 1-beta (IL-1β), and an increase in mRNA levels of the forkhead box L2 gene (FOXL2), octamer-binding transcription factor 4 (OCT4), GDF-9, LIF, and stem cell factor (SCF) in ovarian tissues after transplantation [14]. Several factors such as anti-Müllerian hormone (AMH), mouse vasa homolog, GDF-9, bone morphogenic protein 15 (BMP15), and hyaluronic acid synthase were found to be increased after hAMSC transplantation. These findings support hAMSC regenerative properties in the ovary [15]. Xiao et al. demonstrated that transplantation of hAMSC in mice with POI led to sustained healthy follicle growth, a lower rate of follicle atresia, and restored fertility [16]. These preliminary findings support a role for hAMSC in the treatment of infertility in the setting of POI; however, their efficacy and safety in clinical applications remain to be proven.

### Umbilical Cord Stem Cells

The umbilical cord is another abundant source of MSC; umbilical cord mesenchymal stem cells (UCMSC) are multipotent, non-hematopoietic progenitor cells that can differentiate into multiple cell lines [30, 31]. Mohamed et al. showed that administration of UCMSC into the ovaries of chemotherapy-induced POI mice results in resumption of ovarian function and fertility potential [17]. After therapy, there was a significant decrease in FSH levels, an increase in serum *AMH* levels, resumption of ovulation, and pregnancy followed by the delivery of pups. Thus, treatment with UCMSC in mice with POI resolved both endocrine and exocrine functions of the ovary. Yang et al. demonstrated that the reproductive effects of UCMSC may be due to an increase in ovarian angiogenesis that promotes granulosa cell proliferation and follicle growth and development [18]. Ding et al. transplanted UCMSC on a collagen scaffold into murine ovaries. Activation of primordial follicles occurred via forkhead box class O 3a (FOXO3a) and forkhead box protein O1 (FOXO1), transcription factors that play an important role in folliculogenesis [19]. After 6 h of co-culture with collagen/UCMSC, most primordial follicles were activated. The same study examined the effect of UCMSC with or without collagen in 14 women with POI. In one group, UCMSC were applied alone and in the other, the collagen scaffold was used with UCMSC. One patient from each group was able to achieve clinical pregnancy. These results provide initial evidence of the ability of UCMSC to restore fertility in women with POI. This technique seems promising but further trials with a larger group size need to be done to assess its clinical efficacy and safety.

### Placenta-Derived Stem Cells

Compared to other types of stem cells, human placenta-derived mesenchymal stem cells (hPMSC) are easily accessible. These multipotent and non-hematopoietic progenitor cells have high differentiation and proliferation potential. Yin et al. demonstrated ovarian function restoration in autoimmune-mediated POI mice by intravenous application of hPMSC through regulation of T cells [32]. They observed increased production of cytokines such as transforming growth factor beta (TGF-β), which suppress inflammation and contributed to the recovery of ovarian function. They also noted a concomitant decrease in interferon gamma (IFN-γ), which inhibits ovulation and promotes follicular atresia. Two weeks after hPMSC therapy, the number of estrous cycles was increased, as well as estrogen production, along with a decrease of FSH, thus confirming recovery of ovarian endocrine function. Additionally, they described an increase in blood flow and a decrease in apoptosis of granulosa cells in the mice after hPMSC transplantation. In a subsequent study, this same group demonstrated restoration of ovarian function by activation of the phosphatidylinositol 3-kinase (PI3K)/Akt signaling pathway through T cell regulators. This pathway plays an important role in folliculogenesis and the survival, loss, and activation of primordial follicles in the ovary [33–35]. These preliminary studies show encouraging results; however, the efficacy in humans needs to be tested for further clinical application.

### Human Menstrual Blood Stem Cells

Menstrual-derived stem cells (MenSC) show multipotent and highly proliferative features similar to other mesenchymal stem cells [36]. MenSC express MSC markers and grow and adhere to culture surfaces in vitro [37]. Wang et al. demonstrated that intravenous injection of MenSC can repair ovarian injury and improve ovarian function by stimulating cell proliferation. MenSC transplantation led to an increase in hormone levels such as estrogen, inhibin A, inhibin B, and AMH and hormone receptors such as FSHR. An increase in ovarian size was also noted. The reparative effects of these cells are thought to be mediated through paracrine mechanisms and expression of high levels of fibroblast growth factor 2 (FGF2) [20]. MenSC transplantation may provide an effective and novel method for the treatment of POI. These preliminary studies show reassuring results; however, limited research has been done in this area. We encourage further basic and human studies to build on these initial results for future clinical application.

### Adipose Stem Cells

Adipose-derived stem cells (ASCs) are easy to harvest in high numbers. They can be obtained from subcutaneous adipose tissue in the arm, thigh, or abdomen, as these areas tend to have higher numbers of fat cells [38, 39]. Sun et al. demonstrated that injection of ASCs directly into the mice ovaries or intravenous injection led to an increase in ovarian angiogenesis and a decrease in granulosa cell apoptosis [40]. Mashayekhi et al. performed adipose stem cell isolation from abdominal tissue on 9 women, and after isolation of the ASCs, cells were injected transvaginal; however, they performed laparoscopic transplantation on 2 patients due to technical difficulties. A total of 4 patients demonstrated return in their menstruation cycles and 4 patients had a decrease in FSH levels [41]. This method seems to be a feasible, safe, and innovative therapeutic approach to POI, though few studies have been reported in the literature. Additional basic and human studies are needed to achieve a better understating about the clinical applicability of ASCs for treatment of POI.

### Bone Marrow Stem Cells

Bone marrow stem cells (BMSC) are a type of adult stem cell with low immunogenicity. Similar to other stem cells, they have proliferative and pluripotent potential and under appropriate conditions, they differentiate into bone, cartilage, and adipocytes. Furthermore, BMSC are easy to isolate and grow in vitro. Their role in enhancing the function of endogenous cells and regulating the immune response makes them ideal agents for the repair of tissue damage [42, 43]. Mohamed et al. demonstrated in 2018 that human BMSC are a promising treatment for chemotherapy-induced POI in mice. After BMSC transplantation, they found a significant increase in estrogen, AMH, and inhibin A levels and FSHR expression (*P*<.0001). Treated animals also showed a 100% mating rate [21]. Further studies indicated that the reproductive effect of BMSC is linked to their secretome, which is rich in bioactive factors [44]. Park Soo et al. showed that the BMSC secretome induces proliferation of endothelial cells and enhances angiogenesis. Forty-two angiogenesis gene markers, such as transforming growth factor alpha (TGF-α), epidermal growth factor (EGF), vascular endothelial growth factor-A (VEGFA), and angiopoietin-2 (ANGPT2), were upregulated by the BMSC secretome. TGF-α, C-C motif chemokine 11 (CCL11), leptin (LEP), and EGF are involved in the PI3K/Akt pathway [45]. Igboeli et al. performed autologous bone marrow mesenchymal stem cell transplantation in two women with POI. The cells were obtained, concentrated, and resuspended into 4 mL of bone marrow nucleated cell concentrate. Afterwards, they injected them into one ovary via a laparoscopic approach. Treatment resulted in an increase in baseline estrogen levels along with amelioration of menopausal symptoms. Both patients reported one episode of menstrual bleeding 7 months after transplantation [46]. Edessy et al. transplanted autologous BMSC into the ovary through laparoscopic surgery in 10 women with POI. Two patients regained their menstrual cycle and one achieved a successful pregnancy followed by the delivery of a healthy infant [47]. Gupta et al. published a case report in which a perimenopausal woman achieved a successful pregnancy 8 weeks after transplantation of BMSC [23]. In a study of 30 women with POI, Gabr et al. administered granulocyte colony-stimulating factor (G-CSF) followed by isolation of autologous BMSC, which were injected into the ovarian artery under laparoscopic guidance. They reported one successful pregnancy and an overall improvement in hormonal profile in 86.7% of the women treated. Spontaneous ovulation occurred in 18 women, while 3 underwent in vitro fertilization [48]. Herraiz et al. reported their study of a group of 15 women with poor ovarian response who underwent subcutaneous administration of 10 mg/kg/day of G-CSF for 5 days to promote stem cell mobilization to peripheral blood, followed by stem cell collection by apheresis. Circulating levels of stem cells as assessed by CD34 in peripheral blood were ≥10 cells/μL. The cells were delivered through intraarterial catheterization to the ovarian artery and led to an improvement in follicle count in 81.3% of women, a 33.3% pregnancy rate, 5 pregnancies, and 3 live births [24]. Similar to other types of stem cells, BMSC or the BMSC secretome may be utilized in the treatment of POI. Notable results have been proven in humans, and we believe these results may bring hope to many patients in the future. However, larger clinical trials are needed to confirm these findings.

## In Vitro Activation

The gonadotropin-independent mechanisms involved in the early phases of folliculogenesis encompassing recruitment of primordial follicles, their activation, and transformation into antral follicles remain unclear. Several cellular signaling pathways have been linked to follicle growth. One of these pathways is the Hippo signaling pathway, which constrains follicle growth. Disruption in this pathway has been linked with early activation of the dormant primordial follicles, leading to greater preantral follicle growth. Several intracellular signaling components appear to be involved, such as phosphatase tensin homolog (PTEN) and mTOR [49]. Alteration in these pathways has been shown to cause cellular dysfunction and overgrowth [50]. The PI3K/PTEN/Akt and tuberous sclerosis complex (TSC)/mTOR signaling pathways are known to be critical regulators in meiotic maturation of oocytes, survival, activation, and quiescence of primordial follicles, along with granulosa cell proliferation and differentiation. Changes in one of these pathways can lead to infertility through disruption of follicle maturation and ovulation; in POI, for example, the ovarian cortex contains a large number of follicles stalled at the primordial stage because folliculogenesis is impaired [51]. Kawamura et al. performed laparoscopic surgery in order to isolate the ovarian cortex. After obtaining this tissue, they fragmented the ovarian cortex into 1-2 mm^2^ cubes and proceeded to incubate them with Akt stimulators for 2 days. Through a second laparoscopic surgery, these fragments were autografted beneath the serosa of the fallopian tube. The women were followed weekly or biweekly with transvaginal ultrasonography to monitor follicular growth, which was seen in eight women. When follicles reached the antral stage, as defined by a diameter greater than 5 mm on transvaginal sonography, daily recombinant (r)-FSH treatment was initiated. When follicles reached a diameter greater than 16 mm, an injection of human chorionic gonadotropin (hCG) was administered, simulating the LH surge and initiating oocyte maturation. Transvaginal ultrasound-guided oocyte retrieval was performed 36 h after hCG injection, and a mature oocyte was collected in five of the eight women. Intracytoplasmic sperm injection (ICSI) was then performed with the partner’s sperm, followed by embryo transfer. Two of the five women achieved pregnancy [22]. In 2020, this approach was slightly modified to a more simplistic and less invasive procedure. Eleven women with poor ovarian response or decreased ovarian response were recruited. They were treated with estrogen to suppress FSH and LH levels. Once LH levels were less than 10 mIU/mL, estrogen and progesterone were administered for 10-14 days, after which partial or complete unilateral oophorectomy was performed. The ovarian cortex was isolated and fragmented, then autografted into the contralateral ovary during the same procedure. After the surgical procedure, women were given higher doses than the usual of controlled ovarian hyperstimulation medications. They were followed for 1 year to assess hormone levels and monitor follicle growth by ultrasound imaging. rFSH was used to induce follicular growth; once the dominant follicle reached 14-18 mm in diameter, a trigger shot was administered. Patients were then given the option to attempt natural conception, artificial insemination, or oocyte retrieval followed by embryo transfer. Four patients achieved pregnancy, and four had cryopreserved embryos [52]. This approach is a new strategy in fertility treatment that enables women with POI to conceive using autologous gametes. Improvement in the technique and the positive outcomes hold promise for future clinical application; however, more data in a larger number of women are needed to confirm the efficacy of this treatment.

## Platelet-Rich Plasma

Platelet-rich plasma (PRP) treatment has been described in various clinical settings in regenerative medicine. Autologous PRP is derived from an individual’s whole blood that has been centrifuged to remove red blood cells. The remaining plasma has 5-fold to 10-fold higher concentrations of growth factors than whole blood. These growth factors—including platelet-derived growth factor (PDGF), VEGF, and TGF-β [53, 54]—have been found to promote physiologic healing responses by researchers across multiple specialties, such as dentistry, dermatology, urology, and gynecology [55]. Upon platelet activation, these factors serve as chemo-attractants for stem cells, macrophages, and neutrophils, all of which contribute to the post-translational modification of approximately 1500 bioactive factors [56]. Several animal and human studies have provided insights into the molecular function and structure of platelets [57–59]. Nonetheless, the mitogenic, chemotactic, neovascular, and anti-inflammatory properties of PRP can be attributed to the numerous growth factors it contains [53]. These growth factors are involved in cell migration and differentiation as well as proliferation, activation of angiogenesis, and tissue regeneration [60]. Sills et al. reported the first IVF and blastocyte formation by using PRP treatment. Their procedure consisted of injecting 5 mL of calcium gluconate-activated autologous PRP into the ovary by ultrasound guidance. After their procedure, all patients were followed up with serum AMH, estradiol, and FSH levels every 2 weeks. Once restoration of the ovarian reserve was identified, patients proceeded to IVF therapy. Blastocyte formation was achieved in all patients for cryopreservation. One 9-week pregnancy was reported [25]. Further work was done to evaluate the metabolic and neurobehavioral response after the autologous activated PRP therapy in 2019. Their results indicated increasing life quality in women with POI [61]. In 2020, Sill et al. evaluated 182 patients after autologous PRP therapy, and they noted a 25% increase in AMH levels, which is a clear indication of improvement in the ovarian reserve [62]. Pantos et al. and Sfakianoudis et al. have demonstrated promising results after using PRP in women with POI. Their primary outcome was the restoration of menstruation following PRP treatment. Secondary outcomes included improvement in hormone profile as evidenced by decreased FSH and increased AMH levels. Their procedure consisted of collecting 60 mL of peripheral blood and centrifuging it to obtain 8 mL of PRP. This was then injected evenly into each ovary via a transvaginal approach under ultrasonographic guidance [26, 63]. Hsu et al. reported twin pregnancies in one woman after administration of a combination of 4 mL PRP and 1 mL gonadotropin (150 IU rFSH/75 IU rLH). Spontaneous menses occurred on day 25 and 48 after the procedure [27]. Cakiroglu et al. have reported the largest published clinical trial, which includes a total of 510 patients. They injected 2-4 mL of autologous PRP into the ovary by ultrasound guidance. After the procedure, they achieved increased antral follicle counts and serum AMH levels along with decreased serum FSH levels. Out of the 510 patients, 3.9% were able to conceive spontaneously. A total of 477 patients underwent IVF therapy; 23 of them achieved a successful pregnancy [64–66]. Petryk et al. showed that higher platelet concentration (1 × 10^6^ μL) provides long-lasting results. Thirty-eight women with low ovarian reserve underwent a laparoscopic PRP injection procedure; after therapy, 10 pregnancies were reported. A total of 4 out of 10 were achieved spontaneously, and 6 healthy babies were born [67, 68]. Positive results have been reported in the literature.

## Conclusions

POI is a devastating disease for young women who have not yet completed childbearing. The prevalence of this condition is on the rise due to the increasing number of cancer survivors and the delay in childbearing age. Remarkable efforts are being made to activate remnant primordial follicles in the ovary to regain reproductive potential. In this article, we emphasized novel treatment options, including ovarian tissue activation and the use of stem cells, which have been reported in the literature with promising results including live births. More research is needed to elucidate the most effective and safest approach to stimulate primordial follicles in women with POI to support reproductive function.

## Abbreviations

Akt: protein kinase B
ANGPT2: *angiopoetin-2*
AMH: *anti*-*Müllerian* hormone
ASCs: *adipose-derived stem cells*
BMP15: *bone morphogenic protein 15*
BMSC: *bone marrow stem cells*
CCL11: *C-C motif chemokine 11*
DOR: *decreased ovarian reserve*
EGF: *epidermal growth factor*
FGF2: fibroblast growth factor 2
FSH: follicle-stimulating hormone
FSHR: follicle-stimulating hormone receptor
FOXL2: forkhead box L2
FOXO1: forkhead box protein O1
FOX3a: forkhead box class 0 3a
G-CSF: *granulocyte colony-stimulating factor*
GDF-9: *growth differentiation factor-9*
hCG: human chorionic gonadotropin
hAMSC: human amniotic stem cells
hPMSC: *human placenta-derived mesenchymal stem cells*
ICSI: intracytoplasmic sperm injection
IL-1β: interleukin 1-beta
IFN-γ: *interferon gamma*
IGF-1: *insulin growth factor-1*
LEP: *leptin*
LH: luteinizing hormone
LIF: leukemia inhibitory factor
MenSC: *menstrual-derived stem cells*
MSC: mesenchymal stem cells
mTOR: mammalian target of rapamycin
OCT4: octamer-binding transcription factor 4
PDGF: *platelet-derived growth factor*
PI3K: phosphatidylinositol 3-kinase
POF: premature ovarian failure
POI: premature ovarian insufficiency
POR: poor ovarian reserve
PTEN: phosphatase tensin homolog
PRP: *platelet-rich plasma*
rFSH: recombinant follicle-stimulating hormone
rLH: recombinant luteinizing hormone
SCF: stem cell factor
TGF-α: *transforming growth factor alpha*
TGF-β: *transforming growth factor beta*
TNF-α: *tumor necrosis factor alpha*
TSC: tuberous sclerosis complex
UCMSC: umbilical cord mesenchymal stem cells
VEGF: *vascular endothelial growth factor*
VEGFA: *vascular endothelial growth factor-A*
